# 

**Published:** 1839-07

**Authors:** 


### QUARTERLY JOURNAL

OF

### PRACTICAL MEDICINE AND SURGERY

LIBRARY OF THE

Orleans Parish Medical Society

---

EDITED BY

JOHN FORBES M.D. F.R.S.

---

VOL. VIII.

JULY—OCTOBER 1839

LONDON
JOHN CHURCHILL PRINCES STREET SOHO
MDCCCXXXIX.

LONDON:
PRINTED BY C. ADLARD, BARTHOLOMEW CLOSE.

### BOOKS RECEIVED FOR REVIEW.

1. Elements of Medical Jurisprudence. By Theodore R. Beck, M.D. and John B. Beck, M.D. Sixth Edition.—Philadelphia, 1838. 2 vols. 8vo, pp. 670, 743.

2. A System of Anatomy for the use of Students of Medicine. By Caspar Wistar, M.D., late Professor of Anatomy in the University of Pennsylvania. With Notes and Additions by W. E. Horner, M.D. Seventh Edition, entirely remodelled and illustrated by numerous engravings, by J. Pancoast, M.D., Lecturer on Anatomy and Surgery, &c.—Philadelphia, 1839. 2 vols. 8vo, pp. 491, 560.

3. Political Medicine; being the substance of a Discourse lately delivered before the Royal College of Surgeons in Ireland, on Medicine, considered in its relations to Government and Legislation. By H. Maunsell, M.D. — Dublin, 1839. 8vo, pp. 45.

4. A Letter to the Secretary-at-War, on Sickness and Mortality in the West Indies; being a Review of Capt. Tulloch's Statistical Report. By Sir Andrew Halliday, M.D. F.R.S.E., Dep. Inspector of Hospitals. London, 1839. 8vo, pp. 63. 2s.

5. Report of the Surgical Cases and Operations that occurred in the Massachusetts General Hospital, from May, 1837, to May, 1838. By G. Hayward, M.D., Surgeon to the Hospital.—Boston, 1838. 8vo, pp. 32.

6. Topography of Assam. By John M'Cosh, Lecturer on Clinical Medicine in the Medical School, Calcutta.—Calcutta, 1837. 8vo, pp. 166.

7. School Botany; or, an Explanation of the Characters and Differences of the principal Natural Classes and Orders of Plants belonging to the Flora of Europe. By John Lindley, PH.D. F.R.S., Professor of

Botany in University College, London.—London, 1839. 8vo, pp. 218. 6s.

8. Hints to Mothers, &c. By Thomas Bull, M.D., Lecturer on Midwifery. Second edition, greatly enlarged.—London, 1839. Small 8vo, pp. 307. 7s.

9. Practical Observations on Diseases of Women. By William Jones, Surgeon to the Free Dispensary and Infirmary for Women, &c.—London, 1839. 8vo, pp. 226, with plates. 8s.

10. Medical Portrait Gallery. By T. J. Pettigrew, Esq. Part XIV., containing Portraits and Memoirs of Drs. Heberden and Wilson Philip. April, 1839. 3s.

11. Guy's Hospital Reports, No. VIII. 8vo, pp. 262.—London; April, 1839. 6s.

12. Lectures on Diseases of the Eye. By John Morgan, Surgeon to Guy's Hospital.—London, 1839. 8vo, pp. 221. Illustrated by 18 coloured Plates. 18s.

13. Statistical Reports on the Sickness, Mortality, and Diseases among the Troops in the United Kingdom, the Mediterranean, and British America. Prepared from Records of the Army Medical Department, and War-office Returns.—Lond. 1839. Fol. (From Sir J. M'Grigor.)

14. A Practical Compendium of the Materia Medica, adapted to the Treatment of the Diseases of Infancy and Childhood; with numerous Prescriptions. By Alex. Ure, M.D., A.M. An enlarged Edition, containing simple Rules for the Diet and Regimen of Children, and a Glossary of Technical Terms.—London, 1839. 12mo, pp. 241. 5s. 6d.

15. An Experimental Investigation into the Functions of the Eighth Pair of Nerves, or the Glosso-pharyngeal, Pneumogastric, and Spinal Accessory. By John Reid, M.D. (From the Edin. Med. and Surg. Journal, No. 139.)—Edinburgh, 1839. 8vo, pp. 62.

16. Outlines of Human Physiology. By W. P. Alison, M.D. F.R.S., &c. &c. Third Edition.—Edinb. 1839. 8vo, pp. 457. 12s.

17. Mind, and the Emotions considered in Relation to Health and Disease. By William Cooke, M.D., &c.—London, 1839. 8vo, pp. 56.

18. A Series of Anatomical Sketches and Diagrams, with Descriptions and References. By J. Wormald and A. M. M'Whinnie, Esqrs. of St. Bartholomew's Hospital. Part II.—London, 1839. 4to, 4s.

19. Total Abolition of Personal Restraint in the Treatment of the Insane. A Lecture on the Management of Lunatic Asylums and the Treatment of the Insane. Delivered at the Mechanics' Institution, Lincoln, on the 21st June, 1838. With Statistical Tables. By R. G. Hill, House-Surgeon of the Lincoln Lunatic Asylum.—London, 1839. 8vo, pp. 112. 6s.

20. Medical Portrait Gallery. By T. J. Pettigrew, Esq. F.R.S. Part XV. Containing Memoirs and Portraits of Dr. Cheyne and Dr. Sigmond. May, 1839. 3s.

21. An Essay on the Prevalence of Smallpox, and the Evils of Inoculation: addressed to the Members of the Board of Guardians. By J. D. Jeffery, Esq. Surgeon.—London and Sidmouth, 1838. 8vo, pp. 20.

22. Medical Notes and Reflections. By Henry Holland, M.D. F.R.S., &c. Physician Extraordinary to the Queen.—London, 1839. 8vo, pp. 628. 18s.

23. The valvular Structure of Veins, anatomically and physiologically considered; being the Warneford Prize Essay for 1838. By T. C. Roden, Student of the Birmingham School of Medicine.—Oxford, 1839. 8vo, pp. 65.

24. A Treatise on the Nature of Club-foot, and analogous Distortions; including their Treatment both with and without Surgical Operation. Illustrated by a Series of Cases, and numerous Practical Illustrations. By W. J. Little, M.D., Lecturer on Comparative Anatomy at the London Hospital.—London, 1839. 8vo, pp. 276. 12s.

25. A Report, founded on the Cases of Typhoid Fever, or the common continued Fever of New England, which occurred in the Massachusett's General Hospital from 1821 to 1835. By James Jackson, M.D., late Attending Physician in that Hospital.—Boston, 1838. 8vo, pp. 95.

26. Researches on the Development, Structure, and Diseases of the Teeth. By Alexander Nasmyth, F.L.S., F.G.S., M.R.C.S., &c.—London, 1839. 8vo, pp. 165. With numerous Plates. 10s. 6d.

27. The Transactions of the Provincial Medical and Surgical Association. Vol. VII.—London, 1839. 8vo, pp. 574. With Plates.

28. A Popular Treatise on the Kidney; its hitherto unknown Functions and Diseases, in Connexion with the Circulation of Animal Oils, &c. By George Corfe.—London, 1839. 8vo, pp. 304.

29. The Accoucheur; a Treatise on Protracted Natural Labours; Suspended Animation in New-born Infants; and Uterine Hemorrhage after the Birth of the Child. With illustrative Cases. By John Craig, Surgeon, Paisley.—Glasgow, 1839. 8vo, pp. 252.

30. Müller's Elements of Physiology. Translated by W. Baly, M.D. Part V., containing the Senses.—June, 1839. 4s. 6d.

31. Answers to the Objections commonly brought against Vaccination. By John Robertson, Senior Surgeon to the Manchester Lying-in Hospital.—Manchester, 1839. 8vo, pp. 36.

32. Elements of the Practice of Medicine. By Richard Bright, M.D., and Thomas Addison, M.D., Physicians to Guy's Hospital. Vol. I.—London, 1839. 8vo, pp. 613.

